# Central obesity and atherogenic dyslipidemia in metabolic syndrome are associated with increased risk for colorectal adenoma in a Chinese population

**DOI:** 10.1186/1471-230X-10-51

**Published:** 2010-05-27

**Authors:** Chiu-Shong Liu, Hua-Shui Hsu, Chia-Ing Li, Chia-Ing Jan, Tsai-Chung Li, Wen-Yuan Lin, Tsann Lin, Ya-Chien Chen, Cheng-Chun Lee, Cheng-Chieh Lin

**Affiliations:** 1 Department of Family Medicine, China Medical University and Hospital, Taichung, Taiwan; 2 Department of Medical Research, China Medical University and Hospital, Taichung, Taiwan; 3 Department of Pathology, China Medical University and Hospital, Taichung, Taiwan; 4 Graduate Institute of Biostatistics, College of Public Health, China Medical University, Taichung, Taiwan; 5 Department of Healthcare Administration, Asia University, Taichung, Taiwan; 6 Preventive Medicine Center, China Medical University and Hospital, Taichung, Taiwan; 7 Department of Neurology, China Medical University and Hospital, Taichung, Taiwan

## Abstract

**Background:**

Metabolic syndrome (MetS) is composed of cardiovascular risk factors including insulin resistance, obesity, dyslipidemia, and hypertension. Most of the components of MetS have been linked to the development of neoplasm. The purpose of this study was to evaluate the relationship between individual components of MetS and colorectal adenoma.

**Methods:**

The study subjects were recruited from a pool of 4872 individuals who underwent a health check-up examination during the period January 2006 to May 2008. Each participant fulfilled a structured questionnaire. MetS was defined based on the America Heart Association and National Heart Lung Blood Institute criteria. Subjects with history of colon cancer, colon polyps, colitis, or prior colonic surgery were excluded.

**Results:**

A total of 4122 subjects were included for final analysis (2367 men and 1755 women; mean age, 49.6 ± 11.7 years). Of them, MetS was diagnosed in 708 men (29.9%) and in 367 women (20.9%). Among the patients with MetS, 34.6% had adenoma, 31.7% had hyperplastic polyps and 23.3% were polyp-free (p < 0.0001, Chi-square test). The adjusted OR for colorectal adenoma was significantly higher in the subjects with MetS (OR, 1.31, CI: 1.09-1.57). A stronger association between MetS and colorectal adenoma was found in men (OR:1.44, CI:1.16-1.80) than in women (OR:1.04, CI:0.74-1.46). The adjusted OR for adenoma increased as the number of MetS components increased (p for trend = 0.0001 ). When the individual components of MetS were analyzed separately, only central obesity (OR:1.36, CI:1.14-1.63), low HDL cholesterol levels (OR:1.30, CI:1.10-1.54) and high triglyceride levels (OR:1.26, CI:1.04-1.53) were independently associated with colorectal adenoma.

**Conclusions:**

Of the components of MetS analyzed in this study, central obesity and dyslipidemia are independent risk factors for colorectal adenoma. With regard to the prevention of colorectal neoplasm, life-style modification such as weight reduction is worthwhile.

## Background

Metabolic syndrome (MetS), a collection of cardiovascular risk factors including central obesity, high blood pressure, hyperglycemia, impaired glucose tolerance, hypertriglyceridemia, as well as low levels of high density lipoprotein cholesterol, is associated with an increased risk of cardiovascular disease and diabetes [1-3]. Interestingly, most of the components of metabolic syndrome have also been linked individually to the development of cancer [4-6].

Colorectal cancer is the second leading cause of the cancer incidence and the third leading causes of the cancer death in Taiwan [7]. Several studies have found that obesity, dyslipidemia and hyperglycemia are risk factors for colorectal cancer [8-11]. Furthermore, recent epidemiological studies have reported a positive correlation between metabolic syndrome and the presence of colorectal adenoma [12-14]. In order to prevent colorectal cancer, it is crucial to identify the risk factors that are associated with the development of colorectal neoplasia. The aim of this study was to elucidate the relationship between the components of MetS and prevalence of colorectal adenoma in a Chinese population.

## Methods

### Subjects

All the individuals attending China Medical University Hospital for their routine health survey, during the period January 2006 to May 2008, were invited to participate in this study. A total of 5162 subjects were invited. Colonoscopy was one of the health survey package. Each participant was asked to complete a structured questionnaire designed to collect basic demographic data, medical history and lifestyle characteristics. Smoking status and alcohol consumption history were divided into 3 classes as follows: never, former, and current. For women, history about menopause (yes/no), age of menopause, bilateral oophorectomy (yes/no), and hormone replacement history (yes/no), duration of hormone replacement, were also obtained.

All who willing to complete the health survey, age greater than 30 y/o, were included in the study. Participants with a history of colon cancer, colon polyps, colitis, or prior colonic surgery were excluded from the study. Subjects with family history of familial adenomatous polyposis and patients on life-long anticoagulant therapy with risk of biopsy, were also excluded from the study. Of the 4872 patients that were included, 513 did not sign the consent form, 117 did not complete all phases of the examination or did not undergo a polyp biopsy, and 124 were excluded because of a history of colon disease. Therefore, the final study population was 4122 individuals (2367 men and 1755 women; mean age, 49.6 ± 11.7 years).

### Anthrometric index and laboratory tests

Trained staff measured height, waist and hip circumference (measured to the nearest 0.1 cm) and weight (measured to the nearest 0.1 kg). Weight was measured with light clothing. Waist circumference (WC) was taken at the midway point between the inferior margin of the last rib and the superior iliac crest in a horizontal plane. Body mass index (BMI) was calculated as weight (kg) divided by height squared (m^2^). The same staff measured blood pressure in the right arm using an appropriately sized cuff and a standard mercury sphygmomanometer in a sitting position. Blood was drawn with minimal trauma from an antecubital vein in the morning after a 12-hour overnight fast and was sent for analysis within four hours of blood collection. Hs-CRP levels were measured by measured by nephelometry, a latex particle-enhanced immunoassay (TBA-200FR, Tokyo, Japan). The interassay and intraassay CVs were < 2.0% and < 1.9%, respectively. Biochemical markers such as total cholesterol, low-density lipoprotein cholesterol (LDL-C), high-density lipoprotein cholesterol (HDL-C), triglyceride, fasting glucose, creatinine, and uric acid were analyzed by a biochemical autoanalyzer (Beckman Coulter, Lx-20, USA) at the Department of Clinical Laboratory Diagnostics, China Medical University Hospital.

### Metabolic syndrome

MetS was defined clinically, based on the presence of three or more of the following America Heart Association and National Heart Lung Blood Institute (AHA/NHLBI) metabolic syndrome criteria [15]: (1) central obesity (waist circumference >0.9 m in men, and ≥0.8 m in women), (2) high triglyceride level (≥ 1.7 mmol/L or on drug treatment for elevated triglycerides), (3) low HDL-C level (< 0.9 mmol/L in men and < 1.1 mmol/L in women or on drug treatment for reduced HDL-C), (4) high blood pressure (systolic BP≥ 130 mmHg or diastolic BP≥ 85 mmHg or under anti-hypertensive drug treatment in patients with a history of hypertension), (5) high fasting plasma glucose concentration (≥ 5.6mmol/L or on drug treatment for elevated glucose).

### Screening Colonoscopy

Bowel preparation was done with polyethylene glycol lavage (Klean Prep Powder^®^, Helsinn-Birex Pharmaceuticals, Dublin, Ireland) using a standard protocol identical to that used for diagnostic colonoscopy. The screening colonoscopy was performed by a gastroenterologist. The colonoscope was inserted up to ileocecal area under intravenous general anesthesia with dormicum and alfentanil. Colonoscopic findings were categorized as polyp-free; hyperplastic polyp (no potential of cancer change); low grade adenoma; and high grade adenoma. A neoplasm was classified as a high-grade adenoma based on the following features: a diameter of >1 cm, the presence of three or more lesions, a villous component, or high-grade dysplasia [16]. All the specimen were classified following a pathological examination at the Department of Pathology, China Medical University Hospital. It has been qualified by American College of Pathologist.

### Statistical analysis

The data are presented as mean ± standard deviation (SD) unless indicated otherwise. Log transformation was used for variables with significant deviation from normal distribution, followed by assessment by the Kolmogorov-Smirnov test before further analyses. Analysis of variance (ANOVA) was used for comparison of continuous variables. ANOVA with Scheffe's post hoc comparisons was used. Each pair of the two groups' post hoc comparison was statistically significant at α = 0.0036. Chi-square analysis was used to compare the differences in prevalence of colon neoplasia across MetS groups. Multinominal logistic regression analysis was used to estimate the ORs of colon polyps by age, MetS status, smoking and alcohol consumption status. These statistical analyses were performed using the PC version of SAS statistical software (SAS 9.1.3 Service Pack 2, SAS Institute Inc., Cary, NC, USA). A two-sided p-value of less than 0.05 was considered significant.

We certify that all applicable institutional and governmental regulations concerning the ethical use of human volunteers were followed during this research. Ethics approval for patient recruitment and analyzing the data was obtained from the Institutional Review Board of China Medical University Hospital in Taiwan.

## Results

The prevalence of colon polyp and the individual components of MetS in both sexes are shown in Table 1. Overall, 28.7% of the subjects had a central pattern of obesity, 27.8% had high blood pressure, 58.0% had low HDL-C levels, 23.3% had high triglyceride levels, and 27.6% had elevated glucose levels. In the study population, 26.0% of the subjects (708 men (29.9%) and 367 women (20.9%)) had three or more components of metabolic syndrome.

**Table 1:** Prevalence (%) of metabolic syndrome (MetS), its individual components and colon polyps by sex

	Women (n = 1755)	Men (n = 2367)	Total (N = 4122)
MetS (AHA/NHLBI criteria)	20.9	29.9	26.0
Individual components			
Large waist	27.8	29.3	28.7
High BP	22.7	31.5	27.8
Low HDL-C	58.1	57.8	58.0
High triglycerides	13.5	30.7	23.3
Elevated fasting glucose	22.5	31.4	27.6
Number of components present			
0	26.5	18.3	21.8
1	34.3	25.4	29.2
2	18.3	26.4	22.9
3	11.6	19.0	15.8
4	7.6	8.9	8.4
5	1.7	1.9	1.8
Colonoscopic findings			
Polyp-free	80.9	69.4	74.3
Hyperplastic polyp	5.9	10.0	8.2
Low grade adenoma	10.6	16.6	14.1
High grade adenoma	2.6	4.0	3.4

Abbreviations: AHA/NHLBI, America Heart Association and National Heart Lung Blood Institute; BP, blood pressure; HDL-C, high-density lipoprotein cholesterol.

Of the 4122 patients who underwent a screening colonoscopy, 3062 (74.3%) were polyp-free and 1060 (25.7%) had at least one polyp. Among these patients, hyperplastic polyp was diagnosed in 341(8.3%), low-grade adenoma was diagnosed in 580 (14.1%) and high-grade adenoma was diagnosed in 139 (3.4%) subjects (Table 2). As a whole, subjects without colon polyps were younger, had lower BMI values, waist circumference, blood glucose levels, triglyceride levels, blood pressure, and had higher HDL-C levels than those with colon polyps. The prevalence rates of metabolic syndrome and smoking (current or former) were lower in polyp-free subjects than in patients with colon polyps.

**Table 2:** Demographic and clinical characteristics of the subjects according to colonoscopic findings

	Normal (0) N = 3062	Hyperplastic polyp (1) N = 341	Low-grade neoplasm (2) N = 579	High-grade neoplasm (3) N = 140	ANOVA Scheffe's test ^b^
Age (yrs)	48.2 ± 11.5	51.5 ± 10.8	53. 5 ± 11.1	58.2 ± 11.4	0-1,0-2,0-3, 1-3,2-3
Gender (male)*^a^	1641 (53.6)	237 (69.5)	392 (67.7)	95 (67.8)	
Metabolic syndrome*^a^	714 (23.3)	108 (31.7)	201 (34.7)	48 (34.3)	
BMI (Kg/m^2^)	23.7 ± 3.6	24.7 ± 3.3	24.6 ± 3.4	24.1 ± 3.5	0-1,0-2
WC (m)	0.80 ± 0.10	0.84 ± 0.91	0.84 ± 0.96	0.84 ± 0.99	0-1,0-2,0-3
SBP (mmHg)	117.6 ± 15.0	120.5 ± 14.7	121.8 ± 15.4	121.8 ± 15.6	0-1,0-2,0-3
DBP (mmHg)	74.3 ± 9.4	76.7 ± 9.9	76.7 ± 9.3	77.2 ± 8.8	0-1,0-2,0-3
Glucose (mmol/L)	5.33 ± 1.40	5.59 ± 1.69	5.45 ± 1.14	5.50 ± 1.28	0-1
Cholesterol (mmol/L)	5.14 ± 0.99	5.14 ± 1.09	5.22 ± 1.04	5.23 ± 0.95	NS
Triglyceride (mmol/L)	1.30 ± 1.00	1.44 ± 0.95	1.49 ± 1.06	1.43 ± 1.25	0-2
HDL-C (mmol/L)	1.16 ± 0.34	1.07 ± 0.32	1.07 ± 0.32	1.09 ± 0.33	0-1,0-2
Log CRP^†^	0.08 ± 3.45	0.09 ± 3.52	0.12 ± 3.39	0.12 ± 3.09	0-2,0-3
Smoking status *^a^					
current	589(19.8)	107(32.5)	168(29.9)	39(28.1)	
former	265(8.9)	57(17.3)	66(11.7)	21(15.1)	
never	2121(71.3)	165(50.2)	328(58.4)	79(56.8)	
Alcohol drinking *^a^					
current	835(28.0)	127(38.5)	193(34.5)	49(36.6)	
former	75(2.5)	16(4.8)	33(5.9)	9(6.7)	
never	2067(69.5)	187(56.7)	333(59.6)	76(56.7)	

*p < 0.001 by χ^2 ^test; ^a^: data are presented as n (%); ^b^:Analysis of variance (ANOVA) with Scheffe's post hoc comparisons, each pair of digits represented that two groups' post hoc comparison was statistically significant at α = 0.0036

^† ^: Statistics were tested using the log-transformed values.

Abbreviations: BMI, body mass index; WC, waist circumference; SBP, systolic blood pressure; DBP, diastolic blood pressure; HDL-C, high-density lipoprotein cholesterol; CRP, C-reactive protein;

**Figure 1:**
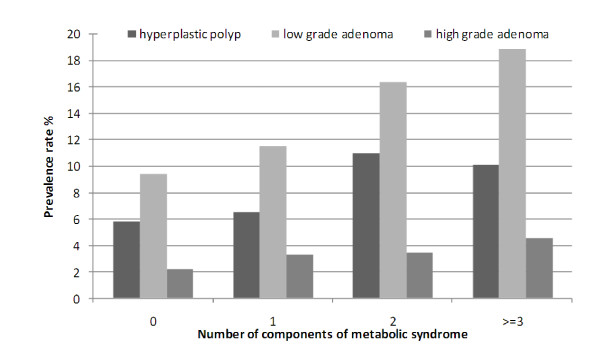
**Prevalence rate (%) of hyperplastic polyp, low-grade adenoma and high-grade adenoma according to the number of components of metabolic syndrome**. The prevalence of both low-grade and high-grade adenoma increased steadily with the cumulative number of components of metabolic syndrome.

The prevalence rate of MetS was 34.6% among subjects with adenoma, 31.7% among those with hyperplastic polyps and 23.3% among poly-free individuals (p < 0.0001, Chi-square test). The prevalence of both low-grade and high-grade adenoma increased steadily with the cumulative number of components of MetS (Figure 1). The adjusted OR for colorectal adenoma was significantly higher in the subjects with MetS (OR, 1.31; CI, 1.09-1.57). The adjusted OR for adenoma increased as the number of components of MetS increased (p for trend = 0.0001 ). This trend was not observed for hyperplastic polyps (Table 3). When the individual components of MetS were analyzed separately, only central obesity, low HDL-C levels and high triglyceride levels were significantly associated with risk for colorectal adenoma (OR:1.36, 1.30, 1.26 respectively, Table 3). The adjusted OR for hyperplastic polyp in the subjects with metabolic syndrome was not significant (OR, 1.26; CI, 0.98-1.61).

**Table 3:** Crude and adjusted odds ratios for colon polyps associated with metabolic syndrome and its components (N = 4122)

	Hyperplastic polyp N = 341		Adenoma (low and high grade) N = 719	
	
	Crude OR (CI)	Adjusted OR (CI)*	Crude OR (CI)	Adjusted OR (CI)*
Metabolic Syndrome				
N = 2944	1.62 (1.28-2.05)	1.26 (0.98-1.61)	1.87 (1.58-2.22)	1.31 (1.09-1.57)
Number of metabolic syndrome components				
1 (N = 1168)	1.17 (0.81-1.69)	1.13 (0.78-1.63)	1.34 (1.02-1.74)	1.23 (0.95-1.61)
2 (N = 914)	1.97 (1.38-2.81)	1.83 (1.28-2.61)	1.86 (1.42-2.43)	1.48 (1.13-1.94)
3 (N = 702)	1.99 (1.36-2.92)	1.45 (0.97-2.16)	2.54 (1.93-3.35)	1.61 (1.21-2.14)
> = 4 (N = 476)	2.45 (1.63-3.67)	2.06 (1.35-3.14)	2.66 (1.97-3.59)	1.70 (1.24-2.34)
Metabolic syndrome components				
Large waist (N = 1179)	1.36 (1.06-1.72)	1.22 (0.96-1.56)	1.64 (1.38-1.94)	1.36 (1.14-1.63)
High BP (N = 1376)	1.61 (1.28-2.02)	1.29 (1.00-1.65)	1.68 (1.42-1.98)	1.08 (0.90-1.30)
Low HDL-C (N = 2389)	1.49 (1.17-1.91)	1.44 (1.14-1.82)	1.33 (1.12-1.59)	1.30 (1.10-1.54)
High triglyceride (N = 1030)	1.40 (1.09-1.79)	1.25 (0.97-1.62)	1.47 (1.23-1.76)	1.26 (1.04-1.53)
High glucose (N = 1149)	1.57 (1.24-1.99)	1.23 (0.96-1.58)	1.58 (1.33-1.88)	1.09 (0.91-1.31)
C-reactive protein^a^	1.12 (1.01-1.25)	1.05 (0.94-1.17)	1.17 (1.08-1.26)	1.08 (1.00-1.17)

*adjusted for age, gender, smoking and alcohol consumption status

^a ^not a component of metabolic syndrome, analyzed as continuous variable after log-transformation

Abbreviations: BP, blood pressure; HDL-C, high-density lipoprotein cholesterol.

The association between MetS and colorectal adenoma was higher in men (OR:1.44, CI:1.16-1.80) than in women (OR:1.04, CI:0.74-1.46). No effects of postmenopausal status or hormone replacement therapy on the colorectal adenoma were found in women. As for the individual component of MetS, dyslipidemia and large waist showed significant association with colon adenoma in men, but not in women. Women had different manifestation, that is, high blood pressure and large waist showed significant association with hyperplastic polyp, a non-precancerous lesions (table 4).

**Table 4:** Adjusted* ORs for colon polyps associated with metabolic syndrome (MetS) and its component according to gender

	Women		Men	
	
	Hyperplastic polyp N = 104	Adenoma (low or high grade) N = 232	Hyperplastic polyp N = 237	Adenoma (low or high grade) N = 487
MetS	1.29 (0.80-2.07)	1.04 (0.74-1.46)	1.22 (0.91-1.64)	**1.44 (1.16-1.80)**
Component of MetS				
Elevated glucose	1.11 (0.68-1.81)	1.17 (0.83-1.64)	1.29 (0.95-1.74)	1.08 (0.86-1.36)
Elevated triglyceride	1.38 (0.83-2.32)	0.98 (0.67-1.45)	1.06 (0.78-1.43)	**1.31 (1.04-1.64)**
Large waist	**1.72 (1.10-2.69)**	1.26 (0.92-1.80)	0.99 (0.73-1.36)	**1.33 (1.06-1.67)**
Low HDL-C	1.30 (0.84-2.01)	1.15 (0.85-1.56)	**1.58 (1.18-2.18)**	**1.45 (1.16-1.80)**
High BP	**1.65 (1.02-2.64)**	1.19 (0.85-1.65)	1.18 (0.87-1.59)	1.05 (0.84-1.31)

*adjusted for age, smoking and alcohol consumption status

Abbreviations: BP, blood pressure; HDL-C, high-density lipoprotein cholesterol.

## Discussion

The prevalence of MetS is increasing due to the escalation of obesity and sedentary lifestyle worldwide. The present study confirmed that certain components of MetS increase the likelihood of colorectal adenoma, a premalignant lesion of colorectal cancer.

Previous studies have found that MetS increases the risk of colorectal adenoma by 1.3 to 2 times [12-14,17]. In present study, we addressed the relationship between colorectal adenoma and the various features of MetS. Kim [14] found that only large waist circumference was associated with the development of colon adenoma. Lee [17] and Wang [12] found that BMI and high triglyceride levels were related to the development of colon or rectosigmoid adenoma. We found that large waist circumference and atherogenic dyslipidemia (low HDL-C and high triglyceride) were associated with the development of colorectal adenoma when individual components were analyzed separately. It is the first time to find the relationship of colorectal adenoma and low serum HDL-C level in the literature.

The possible mechanisms by which colorectal neoplasms arise in subjects with MetS comprise inflammation, insulin resistance and oxidative stress [18]. Adipose tissue is now recognized as an endocrine organ rather than a simple fat storage site, and a wide range of inflammatory cytokines is released from adipose tissue, including C-reactive protein, tumor necrosis factor- and interleukin-6 [19,20]. Adipose tissue can produce and release the inflammatory cytokines that are potentially procarcinogenic. The association of colorectal adenoma and the inflammatory cytokines has been postulated [21-23]. In our study, high-sensitivity C-reactive protein level was significantly higher in MetS subjects with adenoma than in MetS subjects without polyps. This could partly explain the possible mechanism of such phenomenon. Two studies have indicated that patients with multiple components of MetS are at increased risk for colorectal cancer mortality compared with the individual components alone. Cluster analysis indicates that the effects of the individual components are additive [11,18]. We also found that the risk for colorectal ademoma increased with the number of components of MetS.

The association between the MetS and colorectal adenoma was stronger for men than for women. Previous studies have reported the same phenomenon [13,17]; however, those did not take menopausal status of women or the use of hormone replacement therapy into account. It is possible that the protective effect of estrogen or progesterone may be masking the true effects of metabolic syndrome on colorectal neoplasms. The Women's Health Initiative Estrogen Plus Progestin trial suggested that hormone replacement therapy reduced invasive colorectal cancer by 44% [24]. However, a follow-up study of women using estrogen alone did not reveal a reduction in colorectal cancer incidence [25]. Gunter et al postulated two pathways that are related to colon cancer; one involving endogenous estradiol, and the other involving obesity and hyperinsulinemia [26]. In this study, we were unable to confirm whether estrogen or progesterone has a protective effect on the development of colorectal adenoma. More research is needed to clarify the reason for the gender difference in the prevalence of colorectal adenoma in patients with metabolic syndrome.

In addition to smoking and alcohol consumption, confounding factors for colon neoplasm include physical activity, and dietary variables, which were not analyzed in our study. Larsen IK et al [27] reveal that smoking and BMI were the most significant risk factors for neoplasia, but adhering to recommendations on diet and physical activity seems also to be important.

In our study, similar distribution of demographic characteristic between participants and non-participants were found, indicating the kind of selection error might be random. In addition, as long as individuals with the MetS and colon polyps have equal probability of having physical check-up as those individuals with MetS but no colon polyps, the detection error will be random. Thus, the biased results in the effect may be toward the null, a lesser threat to validity.

To evaluate the potential misclassification of polyps/adenoma, we measured the concordance rate of the pathologists. The Kendall's Tau-b statistic was 0.978, which meant a very high inter-rater reliability. The possibility of outcome misclassification was minimal. There also exists the possibility of a measurement error. The inter-assay and intra-assay CVs for cholesterol, triglyceride, glucose and HDL-C were < 4% and < 3%, respectively. Such a measurement error is independent of colon polyp, i.e., due to random error, the results in the effect may be toward the null, a lesser threat to validity.

## Conclusions

The present study of Chinese men and women revealed that increased likelihood of colorectal adenoma, a premalignant lesion of colorectal cancer, was associated with MetS. Of them, central obesity and dyslipidemia were independently increased for the risk of colorectal adenoma. With regard to the prevention of colorectal cancer, life-style modification such as weight reduction is worthwhile.

## Competing interests

The authors declare that they have no competing interests.

## Authors' contributions

CSL, HSH, CCL and CCL contributed equally to the design of the study and direction of its implementation, including supervision of the field activities, quality assurance and control. WYL and TL supervised the field activities. CIL, CIJ and YCC carried out the literature review and prepared the Materials and Methods and the Discussion sections of the text. CSL and TCL designed the study's analytic strategy and conducted the data analysis. All authors read and approved the final version of the manuscript.

## Pre-publication history

The pre-publication history for this paper can be accessed here:

http://www.biomedcentral.com/1471-230X/10/51/prepub
